# Isoprenaline modified the lipidomic profile and reduced β-oxidation in HL-1 cardiomyocytes: *In vitro* model of takotsubo syndrome

**DOI:** 10.3389/fcvm.2022.917989

**Published:** 2022-08-22

**Authors:** Ivana Fiserova, Minh Duc Trinh, Moustafa Elkalaf, Lukas Vacek, Marek Heide, Stanislava Martinkova, Kamila Bechynska, Vit Kosek, Jana Hajslova, Ondrej Fiser, Petr Tousek, Jan Polak

**Affiliations:** ^1^Department of Pathophysiology, Third Faculty of Medicine, Charles University, Prague, Czechia; ^2^Department of Cardiology, Third Faculty of Medicine, Charles University and University Hospital Královské Vinohrady, Prague, Czechia; ^3^Department of Physiology, Faculty of Medicine in Hradec Kralove, Charles University, Hradec Kralove, Czechia; ^4^Department of Biochemistry, Cell and Molecular Biology, Third Faculty of Medicine, Charles University, Prague, Czechia; ^5^Department of Food Analysis and Nutrition, University of Chemistry and Technology Prague, Prague, Czechia; ^6^Department of Biomedical Technology, Faculty of Biomedical Engineering, Czech Technical University in Prague, Prague, Czechia

**Keywords:** takotsubo, isoprenaline, cardiomyocyte, lipid, fatty acid, mitochondria, metabolism

## Abstract

Recent studies have suggested a pathogenetic link between impaired mitochondria and Takotsubo syndrome (TTS), which is closely connected with catecholamine overstimulation, poor outcomes, and changes in lipid metabolism. We investigated the changes in lipid metabolism at the level of fatty acid β-oxidation and changes in the intracellular lipidomic spectrum. The immortalized cell line of HL-1 cardiomyocytes was used in this study as an established *in vitro* model of TTS. The cells were exposed to the non-selective β-agonist isoprenaline (ISO) for acute (2 h) and prolonged (24 h) periods. We investigated the impact on mitochondrial adenosine 5’-triphosphate (ATP) production and β-oxidation using real-time cell metabolic analysis, total lipid content, and changes in the lipidomic spectrum using high-performance liquid chromatography (HPLC) and mass spectrometry. Furthermore, modifications of selected lipid transporters were determined using real-time – polymerase chain reaction (RT-PCR) and/or Western blot techniques. By choosing this wide range of targets, we provide a detailed overview of molecular changes in lipid metabolism during catecholamine overstimulation. The present study demonstrates that acute exposure to ISO decreased ATP production by up to 42.2%, and prolonged exposure to ISO decreased β-oxidation by 86.4%. Prolonged exposure to ISO also increased lipid accumulation by 4%. Lipid spectrum analysis of prolonged exposure to ISO showed a reduced concentration of cardioprotective and an increased concentration of lipotoxic lipid molecules during long-term exposure. Decreased lipid utilization can lead to higher intracellular lipid accumulation and the formation of lipotoxic molecules. Changes in the lipid spectrum can induce pathophysiological signaling pathways leading to cardiomyocyte remodeling or apoptosis. Thus, changes in lipid metabolism induced by excessive doses of catecholamines may cause TTS and contribute to a progression of heart failure, which is at increased risk after a TTS episode.

## Introduction

Takotsubo syndrome (TTS) is a heart disease characterized by temporary left ventricular dysfunction and presents as acute coronary syndrome despite normal angiographic findings of coronary arteries (1). Recent studies suggest that catecholamine overstimulation plays an important role in the development of TTS (2–4). Recent data suggest transient microvascular and endothelial dysfunction leading to impaired contractility (5). TTS is typically characterized by temporary regional akinesia of the apical segments of the left ventricle; other segments of the myocardium may also be affected. Recent data indicates that patients who suffer TTS have a similar risk of developing heart failure as patients who suffer a myocardial infarction (6). TTS is not associated with significant coronary artery obstruction (in contrast to myocardial infarction) (7), and several studies have suggested that transient cardiomyocyte dysfunction occurs due to overexposure to catecholamines with subsequent impairments in intracellular signaling and/or metabolic pathways (8–10). Nevertheless, we do not entirely understand the exact pathophysiological mechanisms that induce TTS, and appropriate targeted treatment is unavailable.

Excessive catecholamine stimulation was shown to induce cardiomyocyte oxidative stress, causing the formation of reactive oxygen species (ROS) (11–15), with subsequent mitochondrial damage expressed as reduced fatty acid β-oxidation, limited movement through the tricarboxylic acid cycle, and decreased adenosine 5′-triphosphate (ATP) production (16, 17). Notably, an increased presence of ROS was detected in human TTS patients on endomyocardial biopsy (18). Limited β-oxidation might lead to lipid accumulation and induce lipotoxicity, as demonstrated in the TTS mouse model (9), where levels of potent signaling lipid molecules, such as diacylglycerol (DAG), increased. Independent studies have shown that elevated intracellular DAG levels modify multiple signaling pathways, including activation of protein kinase C (PKC) and mitogen-activated protein kinase (MAPK) (19, 20), culminating in cardiac remodeling (21, 22). Furthermore, other lipid molecules, e.g., fatty acids, can accumulate inside cardiomyocytes (9, 23–25), for example, lysophosphatidylcholines (LPCs) originating from membrane phospholipids *via* the action of phospholipase A2. Accumulated lipid molecules operate as potent signaling regulators through the activation of multiple intracellular pathways, including protein kinase C and cAMP (through a G protein-dependent pathway), inducing arrhythmias and contributing to the formation of atherosclerotic plaques (26–28) or left ventricular systolic dysfunction (29, 30). In contrast, reduced lipid uptake of left ventricular apical segments in human TTS patients (31, 32) and reduced amounts of long-chain fatty acids in left ventricular cardiomyocytes in a rat model (8) were also reported, demonstrating the complexity of the changes in lipid metabolism regulation in the context of TTS and justifying further investigation.

Only a few studies have addressed changes in specific lipid molecules during TTS, but none have provided extensive information about the lipidomic profile during TTS in the context of mitochondrial damage and metabolic changes.

In this study, we focused on mitochondrial bioenergetics and metabolism (e.g., the production of ATP from fatty acid oxidation), changes in the lipidomic profile, and the gene and protein expression of essential fatty acid transporters, i.e., differentiation Cluster 36 (CD36), long-chain fatty acid transport protein (FATP4), and carnitine palmitoyl transferase 1b (CPT1b) during catecholamine exposure. Based on the assumptions above, we hypothesize that high doses of isoprenaline will induce the accumulation of lipid molecules, presumably due to functional mitochondrial changes (impaired β-oxidation). To better understand the functional consequences of accumulated intracellular lipids and their potential lipotoxic effects, the lipidomic profile will be determined and analyzed. We employed an established *in vitro* model of adult HL-1 cardiomyocytes (a cell line derived from the AT-1 mouse atrial cardiomyocyte tumor line), which retain the morphological, biochemical, and electrophysiological properties of cardiomyocytes.

## Materials and methods

### Cell culture

The HL-1 immortalized cell line (SCC065, Merck, St. Louis, MO, United States) was cultured in Claycomb medium (51800C, Merck) according to an optimized protocol provided by Dr. Claycomb’s laboratory (33). For all experiments, passages 9–12 were used with at least 80% confluency (approximately four days after seeding). For catecholamine overstimulation, cells were exposed to 0.01, 0.1, 0.5, 1, or 2.5 mM ISO (in Claycomb medium with supplements) for 2 or 24 h.

### Oxygen consumption rate measurement

Mitochondrial functions and fatty acid oxidation of HL-1 cells were assessed using Seahorse XFe24 and XFe96 (Agilent Technologies, Santa Clara, California, United States). Cells were plated on Seahorse XFe24 or XFe96 cell culture microplates 24–72 h before measurement under standard culture conditions described above. Oxygen consumption rate (OCR) was measured *via* the Seahorse XF Cell Mito Stress kit (103015-100, Agilent Technologies) and a Seahorse XFe24 Analyzer following the manufacturer’s protocols for exposure to isoprenaline (0.01, 0.1, 0.5, 1.0, and 2.5 mM), with the addition of Oligomycin (1.5 μM), Carbonyl cyanide-p-trifluoromethoxy phenylhydrazone (FCCP – 0.5 μM), and rotenone/antimycin A (0.5 μM) during measurement. The fatty acid consumption rate was measured using an etomoxir inhibitor, which inhibits the CPT-1 transporter. Cells were exposed to 0.5, 1.0, and 2.5 mM of ISO for 2 or 24 h before measurement. Before the flux assay, cells were starved in a low nutrient Dulbecco’s modified eagle medium (DMEM) medium (2.5 mM glucose, 1.0 mM glutamine, 1.0 mM pyruvate, and 1.0 mM carnitine) and incubated in a CO_2_-free incubator for 2 h together with ISO. Etomoxir was added 20 min after the OCR measurement. Differences in OCR response to respiratory inhibition were used to calculate various mitochondrial parameters (ATP-linked respiration, maximum respiration, proton leak respiration, and fatty acid-linked respiration). Differences in extracellular acidification rate (ECAR) data after CPT1 inhibition by etomoxir were used to calculate anaerobic glycolysis. Data were normalized to the number of cells or basal respiration.

### RNA isolation and quantitative polymerase chain reaction

Total RNA from HL-1 cells was isolated using a High Pure RNA Isolation Kit (11828665001, Roche Diagnostics GmbH, Penzberg, Germany). RNA was quantified by assessing the optical density at 260 and 280 nm (NanoDrop 2000, Thermo Fisher Scientific, Waltham, MA, United States). Extracted RNA was transcribed using a High-Capacity cDNA Reverse Transcription Kit (4368814, Roche Diagnostics). Gene expression was noted using TaqMan probes (Thermo Scientific) of CD36 (Mm01135198_m1), FATP4 (Mm01327405_m1) CPT1b (Mm00487200_m1), TATA box binding protein (TBP, Mm00446971_m1), Glucuronidase beta (GUSB, Mm01197698_m1) and was assessed by Applied Biosystems™ 7500 Fast Real-Time PCR (Applied Biosystems, Boston, United States). TBP and GUSB were used as endogenous controls. Data are presented as the relative gene expression change comparing target genes to the geometric mean expression of endogenous controls using the 2^–Δ^ *^CT^* method.

### Western blot

HL-1 cells were washed with cold phosphate-buffered saline (PBS) and lysed in Tissue Protein Extraction Reagent (78510, Thermo Scientific) buffer. The lysate was centrifuged at 12,000 rpm for 15 min at 4°C. Proteins were separated using 10% Mini-PROTEAN^®^ TGX™ Precast Gels (4561033, Bio-Rad, Hercules, United States) and transferred to polyvinylidene difluoride membranes (1620177, Bio-Rad). Membranes were incubated in EveryBlot Blocking Buffer (12010020, Bio-Rad). Subsequently, membranes were incubated with primary antibodies against CD36 (ab133625, Abcam, Cambridge, United Kingdom), FATP4 (ab199719, Abcam), and CPT1B (ABIN2782633, antibodies-online, Aachen, Germany) at 4°C overnight. The day after, membranes were incubated with secondary antibody (ab97051, Abcam) for 1 h at room temperature. Protein content was normalized to Glyceraldehyde-3-Phosphate Dehydrogenase (GAPDH - D16H11, Cell Signaling, Danvers, United States) for quantification of optical density. Immunoreactive bands were visualized using Radiance chemiluminescent solution (Ac2103, Azure Biosystems, Dublin, United States) and detected using an Azure c300 imaging device (Azure Biosystems).

### Intracellular lipid content and lipidomic analysis

Intracellular lipids were extracted using a chloroform-free lipid extraction kit (ab211044, Abcam) according to the manufacturer’s recommendation. Lipids were quantified using a neutral lipids assay kit (ab242307, Abcam) according to the manufacturer’s protocol. Lipids were normalized to the total protein amount for each sample. For lipidomic analysis, lipids were extracted in a mixture of methyl tert-butyl ether and methanol (10:3). Subsequently, deionized water was added, and the samples were centrifuged (14,000 rpm, 10 min) and evaporated to dryness. The samples were then reconstituted in a mixture of isopropanol, methanol, and water (65:30:5). For the lipidomic analysis, U-HPLC (Infinity 1290, Agilent) was coupled to a high-resolution mass spectrometer with a hyphenated quadrupole time-of-flight mass analyzer (6560 Ion Mobility Q-TOF LC/MS; Agilent, United States); an Agilent Jet Stream electrospray source was employed. An Acquity BEH C18 [1.7 μm, 2.1 mm × 150 mm (Waters, Milford, United States)] was used for chromatographic separation. The conditions of chromatographic separation and mass analyzer settings are described in detail in the online Supplementary material. A QC sample was run every ten samples to assess system stability. The data were processed using the LipidMatch suite (DOI: 10.1186/s12859-017-1744-3, MetaboAnalyst, Edmonton, Canada), which relies on MZMine for feature extraction and custom R scripts for lipid identification using sum normalization, logarithmic transformations, and Pareto scaling.

### Statistical analyses

The results are expressed as boxplots depicting the mean value (horizontal line), interquartile range (IQR) (box), and 1.5 × IQR (whiskers). All technical replicates were averaged. Statistical methods included Student’s unpaired *t*-test (two groups), one-way analysis of variance (ANOVA) with multiple comparisons (three or more groups) using Dunnett’s test, or two-way ANOVA with multiple comparisons (three or more groups with two factors). A *p* < 0.05 was considered to indicate a significant difference between groups. In the figures, *p* < 0.05 is shown with *, *p* < 0.01 is shown with ^**^, *p* < 0.001 is shown with ^***^ and *p* < 0.0001 is shown with ^****^. All data were tested for outliers using the Tukey outliers test. The Shapiro-Wilk test was used to assess normal distributions. All statistical analyses were carried out using MATLAB R2021a (Mathworks, Massachusetts, United States).

## Results

### Exposure to isoprenaline reduced adenosine 5’-triphosphate production and maximal respiration

**FIGURE 1:**
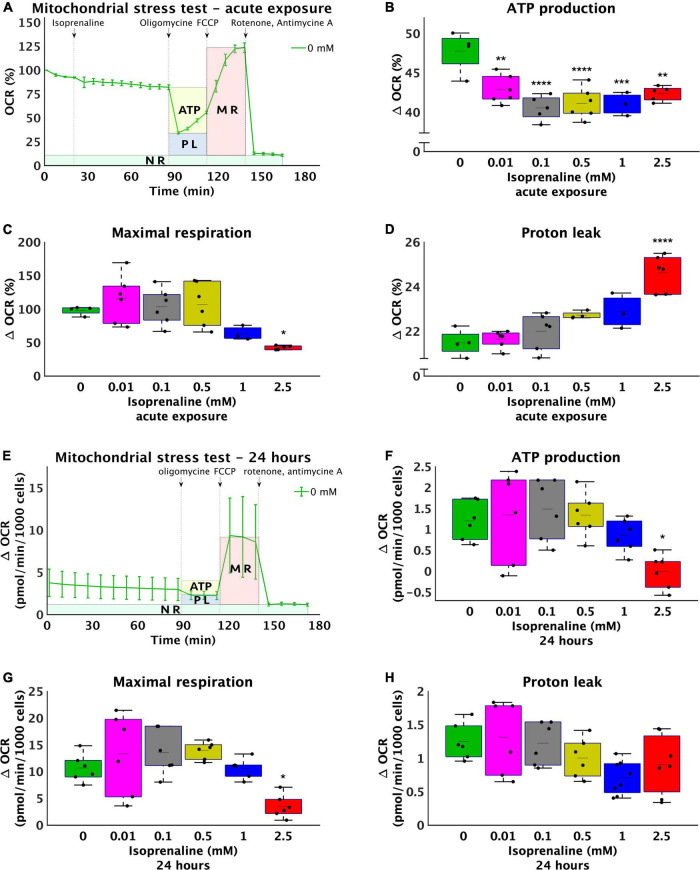
Increasing doses of isoprenaline impair mitochondrial functions. **(A)** Representative graph of the oxygen consumption rate (OCR) of HL-1 cardiomyocytes exposed to different levels of isoprenaline showing the calculation of mitochondrial functions after acute ISO exposure. NR, non-mitochondrial respiration, ATP, ATP production, PL, proton leak, MR, maximal respiration, **(B)** Quantification of ATP production calculated from **(A)**, one-way ANOVA: *p* = 5.83 × 10^–5^. **(C)** Quantification of maximal respiration from **(A)**, one-way ANOVA: *p* = 0.0009. **(D)** Quantification of proton leak from **(A)**, one-way ANOVA: *p* = 2.79 × 10^–7^. **(A–D)** *n* = 3–6 biological replicates per group, one technical replicate, **(E)** Representative graph of the oxygen consumption rate (OCR) of HL-1 cardiomyocytes exposed to different levels of isoprenaline showing the calculation of mitochondrial functions after 24 h of exposure to ISO. **(F)** Quantification of ATP production from **(E)**, one-way ANOVA: *p* = 0.0041. **(G)** Quantification of maximal respiration from **(E)**, one-way ANOVA: *p* = 0.0006. **(H)** Quantification of proton leak calculated from **(E)**, one-way ANOVA: *p* = 0.028. **(E–H)** *n* = 6 biological replicates per group, one technical replicate. Statistical significance was assessed using one-way ANOVA followed by Tuckey multiple comparisons.

The study was initiated by studying the effects of isoprenaline on mitochondrial ATP production, maximal respiration, and proton leakage through exposure of HL-1 cardiomyocytes to 0.01 mM, 0.1 mM, 0.5 mM, 1.0 mM, and 2.5 mM isoprenaline. The calculation of basic mitochondrial function and OCR measurement of control group during acute exposure to ISO is shown in Figure 1A. Mean ATP production (Figure 1B) was reduced in all ISO exposed groups (from 47.8 ± 2.6% - control to 42.9 ± 1.8% – 0.01 mM of ISO, 40.5 ± 1.5% – 0.1 mM of ISO, 41.1 ± 2.0% – 0.5 mM of ISO, 41.0 ± 1.5% – 1 mM of ISO, and 42.4 ± 1% – 2.5 mM of ISO, *p* < 0.01). The highest dose of ISO reduced maximal respiration by 2.3-fold (from 97.9 ± 6.6% – control to 42.4 ± 3.0% – 2.5 mM of ISO, *p* < 0.05); lower doses of ISO did not induce changes in maximal respiration (Figure 1C). Proton leakage tended to be higher with increasing doses of ISO, but only the highest dose of ISO was significantly increased by 1.1-fold (from 21.5 ± 0.6% – control to 24.6 ± 0.8% – 2.5 mM of ISO, *p* < 0.05, Figure 1D). The real-time oxygen consumption rate of all groups is shown in Supplementary Figure 1.

The same experimental design was used on cells exposed to ISO for 24 h – prolonged exposure. The calculation of basic mitochondrial function and OCR measurement of control group during prolonged exposure to ISO is shown in Figure 1E. Mean ATP production (Figure 1F) was absolutely reduced in group exposed to 2.5 mM of ISO, where the mean value of ATP production was very close to zero (1.2 ± 0.47 pmol/min/1000 cells – control to –0.01 ± 0.41 pmol/min/1000 cells – 2.5 mM of ISO, *p* < 0.05). Mean maximal respiration was also reduced in the group exposed to the highest dose of ISO (3-fold 10.67 ± 2.59 pmol/min/1000 cells – control to 3.51 ± 2.17 pmol/min/1000 cells – 2.5 mM of ISO, *p* < 0.05), while lower doses of ISO did not induce changes in maximal respiration (Figure 1G). Proton leak did not significantly change. The real-time oxygen consumption of all groups exposed to ISO for 24 h is shown in Supplementary Figure 1.

### Isoprenaline exposure for 24 h reduced β-oxidation and increased intracellular lipids

**FIGURE 2:**
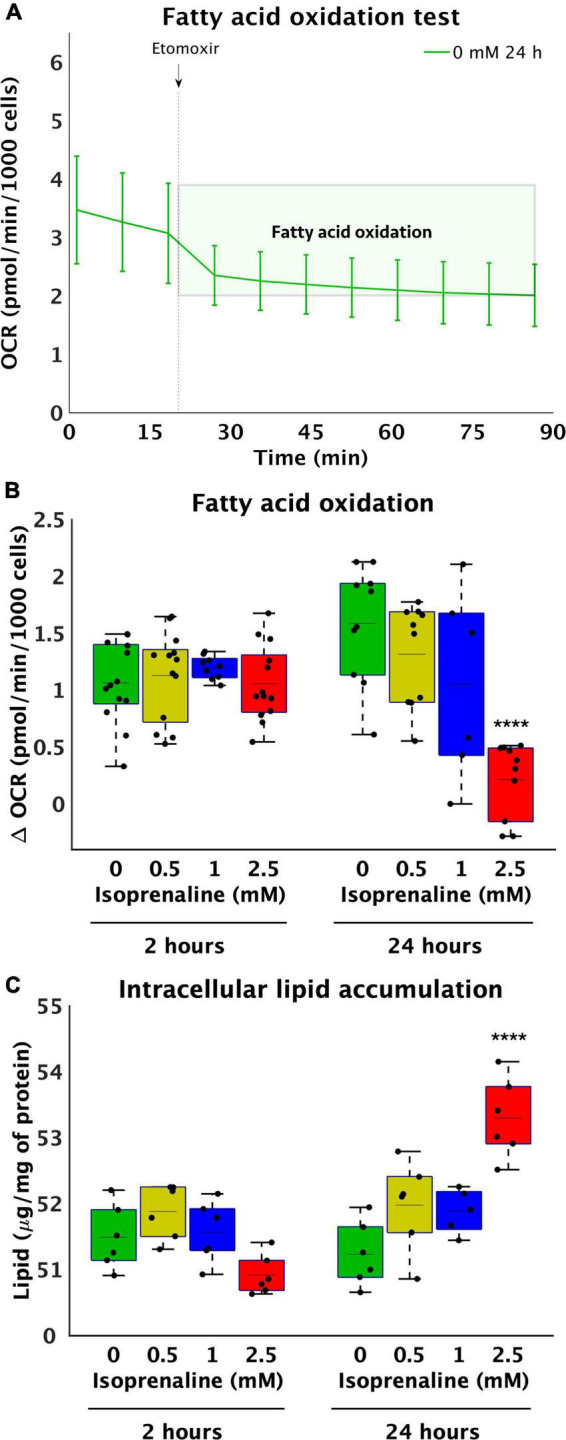
Reduce utilization of medium-length fatty acids and increase lipid accumulation after high-level and long-term exposure to isoprenaline. **(A)** Representative graph of the oxygen consumption rate (OCR) of HL-1 cardiomyocytes before and after 40 μM etomoxir inhibition. **(B)** Quantification of fatty acid oxidation calculated from **(A)**. Statistical significance was assessed using two-way ANOVA (effect of concentrations: *p* = 4.53 × 10^–7^, time: *p* = 0.5643, interaction: *p* = 1.76 × 10^–6^) followed by Sidakholm multiple comparisons. **(A,B)** *n* = 12 biological replicates per group, one technical replicate, **(C)** Intracellular lipid accumulation in HL-1 cardiomyocytes expose to 0 mM, 0.5 mM, 1.0 mM, and 2.5 mM levels of isoprenaline for 2 h and 24 h (effect of concentrations: *p* = 7.03 × 10^–5^, time: *p* = 0.0043, interaction: *p* = 2.13 × 10^–7^). *n* = 6 replicates per group, 2 technical replicates.

To further explore metabolic pathways contributing to the observed decrease in ATP production, the level of β-oxidation was determined by inhibiting CPT1 with etomoxir for 2 and 24 h, while the rate of oxygen consumption before and after the addition of etomoxir was measured using a Seahorse Analyzer XFe24 (Figure 2A). During acute exposure to all concentrations of isoprenaline, no changes in β-oxidation were observed. However, prolonged exposure to 2.5 mM ISO reduced β-oxidation by 7.6-fold (from 1.59 ± 0.51 pmol/min/1000 cells – control to 0.21 ± 0.33 pmol/min/1000 cells – 2.5 mM of ISO, *p* < 0.0001), as summarized in Figure 2B. The real-time rate of fatty acid oxygen consumption in all groups exposed to ISO for 24 h is shown in Supplementary Figure 2. At the same time, the level of glycolysis was calculated during post-CPT1 inhibition from the ECAR data. These results showed that acute ISO exposure indeed increased glucose flux by 2.1-fold at the highest dose of ISO (from 0.09 ± 0.05 pH*10^–3^/min/1000 cells – control to 0.19 ± 0.04 pH*10^–3^/min/1000 cells – 2.5 mM of ISO, *p* < 0.0001), while prolonged ISO exposure did not have a significant effect on glucose flux.

Reduced fatty acid oxidation after 24 h raises the question of whether lipid accumulation occurs within cardiomyocytes. No changes were observed after 2 h of ISO exposure, which was expected based on unchanged β-oxidation. However, changes were observed after 24 h of treatment at the highest dose (2.5 mM) of ISO, which was increased by 1.04-fold (from 51.23 ± 0.49 μg/mg of protein – control to 53.30 ± 0.60 μg/mg of protein, *p* < 0.0001, Figure 2C), correlating with lower β-oxidation.

### The effect of isoprenaline on lipid transporter genes and protein expression

**FIGURE 3:**
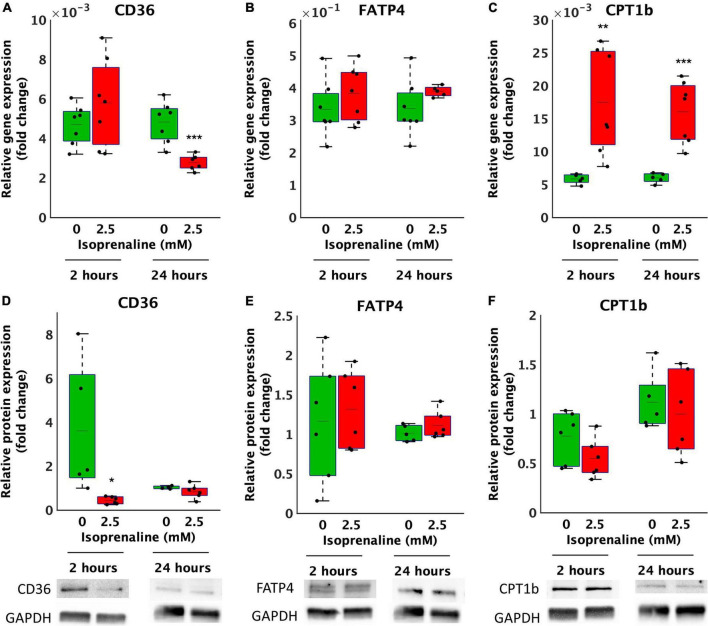
Altered gene and protein expression of lipid transporters in HL-1 cardiomyocytes exposed to isoprenaline. **(A–C)** qPCR analysis of HL-1 cardiomyocytes exposed to 0.0 mM and 2.5 mM levels of ISO for 2 h (green boxes) and 24 h (red boxes). Statistical significance was assessed using a two-tailed *t*-test. *N* = 6 replicates per group. All values shown for the PCR analyses are relative to the housekeeping gene TBP and GUSB. *n* = 6 replicates per group, 3 technical replicates, **(D–F)** Western blot analyses of HL-1 cardiomyocytes exposed to 0.0 mM and 2.5 mM levels of ISO for 2 h (green boxes) and 24 h (red boxes). Statistical significance was assessed using a two-tailed *t*-test. All values shown for the PCR analyses are relative to the loading control GAPDH. *n* = 6 replicates per group, 2 technical replicates.

Decreased β-oxidation due to ISO exposure may be caused to reduced transport of FA by transport proteins. Thus, the relative gene and protein expression of the most important lipid uptake regulators *CD36* and *FATP4* located on the cytoplasmic membrane and *CPT1b* on the mitochondrial membrane in cardiomyocytes exposed to 2.5 mM ISO for 2 and 24 h was determined. After 2 h of ISO exposure, no changes in the relative gene expression of *CD36* and *FATP4* were observed (Figures 3A,B). However, the relative gene expression of *CPT1b* increased by 3-fold after 2.5 mM ISO exposure (17.5 × 10^–3^ ± 7.9 × 10^–3^ vs. 5.9 × 10^–3^ ± 7.3 × 10^–4^, *p* < 0.01, as summarized in Figure 3C). In contrast, 24-h exposure to 2.5 mM ISO decreased *CD36* gene expression by 1.7-fold (2.8 × 10^–3^ ± 3.9 × 10^–4^-fold vs. 4.8 × 10^–3^ ± 1.0 × 10^–3^-fold, *p* < 0.0001, Figure 3A) and increased the relative gene expression of *CPT1b* by 2.7-fold (16.0 × 10^–3^ ± 4.7 × 10^–3^-fold, vs. 6.0 × 10^–3^ ± 7.5 × 10^–3^-fold, *p* < 0.0001, Figure 3C); the same was true for 2 h of exposure. There were no changes in the relative gene expression of *FATP4* after 24 h of exposure to ISO.

At the relative protein expression level, after 2 h of ISO exposure, CD36 decreased by 8.2-fold (from 3.6 ± 3.0-fold – control to 0.4 ± 0.2-fold, *p* < 0.05, Figure 3D). After 24 h of ISO exposure, no changes were observed. Changes in the relative protein expression of *FATP4* and *CPT1B* at both time points were also not observed (Figures 3E,F).

### Isoprenaline exposure for 24 h modified the lipidomic profile

**FIGURE 4:**
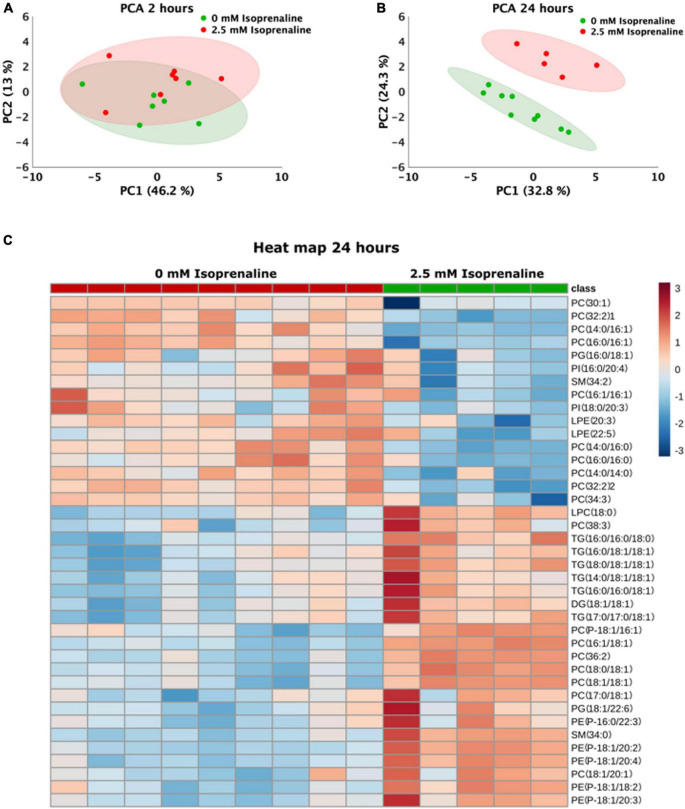
Changes in the lipidomics profile induced by high-level and long-term exposure to isoprenaline. **(A,B)** Principal Component Analyses showed that prolonged exposure of HL-1 cardiomyocytes to 2.5 mM of isoprenaline promotes a distinct shift of the lipidomic profile compared to 2 h of exposure. **(C)** Clustering result of statistically significant molecules of lipids in the HL-1 cardiomyocytes exposed to 2.5 mM of isoprenaline for 24 h. *n* = 5–9 biological replicates per group, one technical replicate.

The lipidomic analysis identified 164 lipid compounds in cells. The principal component analysis (PCA) after a 2-h exposure to 2.5 mM ISO revealed cluster overlapping, indicating no significant changes in the lipid profile after 2 h of ISO exposure (Figure 4A). After 2 h of ISO exposure, five lipids were elevated: 2 lysophosphatidylcholines, 2 phosphatidylethanolamines, and phosphatidylglycerol (Table 1). The lipidomic profiles after 24 h of exposure to 2.5 mM ISO showed significant separation from control conditions, as shown in the PCA (Figure 4B). Profound changes in the lipidomic profile were detected after 24 h of ISO exposure, where 39 lipid molecules were differentially affected. Among the identified lipids, the most significant fold change was seen in phosphatidylethanolamines [PE (P-18:1/20:4) – 3.88-fold]. Diacylglycerol (DG), triacylglycerols (TG), and most phosphatidylcholines (PC) also showed significant increases. All lysophosphatidylethanolamines (LPE) were reduced. The data are shown as a heatmap (Figure 4C). Significant changes are summarized in Table 2. All data for 2 and 24-h exposure are summarized in a Supplementary material (Figure 3 and Tables 1, 2)).

**TABLE 1:** Lipidomic changes after 2 h of isoprenaline (ISO) exposure.

Category	Subclass	Common name	Fold change	FDR p-value
Glycerophospholipids	Lysophosphatidylcholines	LPC(18:1)	1.44	0.036
		LPC(16:0)	1.33	0.036
	Phosphatidylethanolamines	PE(P-18:1/18:2)	1.33	0.036
		PE(P-18:1/20:4)	2.46	1.2 × 10^–4^
	Phosphatidylglycerol	PG(18:1/22:6)	1.75	0.036

**TABLE 2:** Lipidomic changes after 24 h of isoprenaline (ISO) exposure.

Category	Subclass	Common name	Fold change	FDR p-value
Sphingolipids	Sphingomyelins	SM(34:2)	0.78	3.65 × 10^–4^
		SM(34:0)	2.54	6.66 × 10^–6^
Glycerolipids	Diacylglycerol	DG(18:1/18:1)	1.34	0.014
	Triacylglycerols	TG(16:0/16:0/18:0)	1.35	0.046
		TG(16:0/16:0/18:1)	1.25	0.013
		TG(14:0/18:1/18:1)	1.15	5.17 × 10^–3^
		TG(16:0/18:1/18:1)	1.44	0.042
		TG(18:0/18:1/18:1)	1.78	0.005
		TG(17:0/17:0/18:1)	1.59	0.022
Glycerophospholipids	Lysophosphatidylcholine	LPC(18:0)	1.48	2.49 × 10^–3^
	Lysophosphatidylethanolamines	LPE(22:5)	0.48	0.037
		LPE(20:3)	0.13	0.013
	Phosphatydilcholins	PC(32:2)1	0.73	2.1 × 10^–3^
		PC(14:0/16:1)	0.74	2.84 × 10^–3^
		PC(16:1/16:1)	0.66	0.032
		PC(32:2)2	0.71	1.14 × 10^–5^
		PC(16:0/16:1)	0.62	4.41 × 10^–3^
		PC(38:3)	1.29	0.043
		PC(16:1/18:1)	1.58	2.1 × 10^–3^
		PC(17:0/18:1)	1.23	0.012
		PC(14:0/16:0)	0.54	3.3 × 10^–5^
		PC(14:0/14:0)	0.77	1.9 × 10^–3^
		PC(34:3)	1.42	1.69 × 10^–3^
		PC(30:1)	0.6	3.65 × 10^–4^
		PC(16:0/16:0)	0.57	2.53 × 10^–3^
		PC(36:2)	1.53	0.005
		PC(18:0/18:1)	1.3	0.023
		PC(P-18:1/16:1)	1.69	0.043
		PC(18:1/18:1)	1.39	0.023
		PC(18:1/20:1)	1.27	0.013
	Phosphatidylethanolamines	PE(P-16:0/22:3)	1.27	2.52 × 10^–3^
		PE(P-18:1/20:2)	2.74	3.04 × 10^–7^
		PE(P-18:1/20:3)	1.65	5.56 × 10^–4^
		PE(P-18:1/18:2)	2.16	1.69 × 10^–3^
		PE(P-18:1/20:4)	3.88	1.75 × 10^–5^
	Phosphatidylglycerols	PG(16:0/18:1)	0.67	0.01
		PG(18:1/22:6)	1.38	0.022
	Phosphatidylinositols	PI(18:0/20:3)	1.32	0.038
		PI(16:0/20:4)	0.77	0.005

## Discussion

Although takotsubo syndrome is classified as an acute coronary syndrome, its pathophysiological basis involves metabolic changes in cardiomyocytes due to extreme catecholamine stimulation (8) rather than damage to coronary vessels (7). Our study demonstrates that isoprenaline induces mitochondrial damage that directly leads to the formation of lipotoxic molecules. To investigate the effects of acute (2 h) and long-term (24 h) exposure to isoprenaline, we used the adult cardiomyocyte cell line, HL-1, capable of spontaneous contraction with a stable phenotype (9).

The main finding of this study was that prolonged exposure to ISO decreased mitochondrial function, increased total lipid content, and extensively modified the intracellular lipidomic profile with increased levels of potentially lipotoxic molecules. Previous studies have confirmed that all these factors can induce the apoptosis (34) and contractile dysfunction (35–37) observed in TTS.

The study showed that long-term exposure to ISO reduced FA utilization during β-oxidation without affecting the amount of the major FA transporters CD36, FATP4, and CPT1b. In addition, 2- and 24-h exposure to isoprenaline caused reduced ATP production, which may reflect direct mitochondrial damage due to ROS (11) and the reduced fatty acid utilization capacity observed in this work. Moreover, in the case of acute exposure, maximal respiration was inhibited, and proton leakage was increased at the highest isoprenaline concentration. Previous studies have observed that β-adrenergic stimulation by catecholamines induces oxidative stress and ROS production (11) with subsequent disruption of mitochondrial membrane potential leading to increased proton leak (12). Thus, it is very likely that mitochondrial damage due to oxidative stress caused by excessive catecholamine stimulation is also present in this case. Other factors may also influence mitochondrial bioenergetics. Regulation of β-oxidation may also be controlled by intracellular levels of malonyl-CoA, which acts as an allosteric inhibitor of CPT-1. Increased intracellular levels of malonyl-CoA after isoprenaline administration have previously been observed in rats, along with decreased FA oxidation and decreased Krebs cycle intermediates (8). However, increased malonyl-CoA levels were observed in isolated cardiomyocytes (38), indicating differences in the models and doses of isoprenaline used (*in vivo*, isolated cardiomyocytes, or HL-1 cardiomyocytes) could play an important role in determining the metabolic response to catecholamines. Decreased β-oxidation and increased lipid accumulation are often associated with contractile dysfunction in the hearts of obese rats (35–37), which could represent a molecular link to reduced cardiomyocyte contraction occurring in TTS.

If we focused on glycolytic pathway, we observed increased glucose flux in acute ISO exposure to highest dose (without changed lipid oxidation), while prolonged exposure did not have significant effect on glucose flux (but reduced lipid oxidation). These results showing metabolic differences induced by acute and prolonged exposure are surprising, as compensation for reduced FA oxidation by increased glucose consumption is unlikely to occur. Similar results for TTS were observed (8). From these results, we can speculate that the catecholamine level is already so high that the glycolytic pathway is also impaired and is no longer able to compensate for the ATP deficit caused by the reduced beta-oxidation pathway. The availability of FA substrates may also be affected by their transport across cellular and mitochondrial membranes.

Our study observed decreased CD36 protein expression after acute exposure without a complementary decrease in β-oxidation. At the same time, no increased lipid accumulation was observed in this group compared to cardiomyocytes with prolonged exposure to isoprenaline. Thus, it is likely that in the case of acute isoprenaline exposure, induction of cardiomyocyte lipid accumulation was counteracted by a decrease in protein expression of the major membrane fatty acid transporter CD36. Increased CPT1b expression was observed in acute exposure relative to gene expression. However, protein levels did not follow trajectories consistent with gene expression, as observed in other studies (39). At the gene expression level, there was a decrease in the CD36 receptor. This mismatch may be explained by increased protein degradation by sumoylation, ubiquitination (40), or epigenetic modifications, including histone acetylation and DNA methylation (41, 42). No changes in CD36, FATP4, and CPT1 protein levels were detected after prolonged exposure, although β-oxidation was reduced at this time. At the gene expression level, there was a decrease in the CD36 receptor. A mismatch between gene and protein expression of the CD36 receptor was evident, which may be explained by the relatively high stability of this protein due to its two transmembrane domains, disulfide bonds, and glycosylation site that protects against proteases (42). The authors suggest that acute and prolonged exposures activate various intracellular signaling cascades and trigger different (metabolic) adaptations in cells. During acute ISO exposure, reduced protein expression of CD36 can be explained by increased rapid ubiquitination, which was observed in cardiomyocyte in other study (43, 44), while the regulation of gene expression plays only a limited role. A different picture was obtained after prolonged isoprenaline exposure, where cellular metabolic adaptations, including significant intracellular lipid accumulation, were observed and could affect the regulation of gene/protein expression, for example, by inhibition of PPARγ transcription factor resulting in decreased gene expression of CD36 (44) with unaffected protein levels. Similar protein and gene expression results have already been observed relative to lipid transporters in animal and cell TTS models (8–45). From the results of the present study, we can deduce that prolonged exposure to ISO (which is associated with greater cellular damage) does not affect the expression of lipid carrier proteins.

Analyses focusing on lipid accumulation and lipidomic spectrum provide the most interesting results of this study. ISO treatment had temporally different effects on total intracellular lipid accumulation. Whereas acute exposure to ISO showed no effect on total lipid accumulation and induced undetectable differences (relative intensities were altered for 5 of 164 lipids identified) by PCA, prolonged exposure slightly increased total lipid amounts and profoundly restructured the lipid spectrum with complete segregation of treatment and control groups according to the PCA analysis. Increased lipid accumulation has already been observed in humans suffering from TTS (9), in mouse and rat models of TTS (9, 46, 47), and in cardiomyocytes derived from pluripotent stem cells (45). The mechanism of this accumulation remains unclear, with a proposed mechanism involving reduced fatty acid export (9) or increased nicotinamide adenine dinucleotide phosphate (NADPH) oxidase (NOX1 and NOX4) activity stimulating fatty acid synthase and thus *de novo* lipogenesis (46). However, our study suggests that the increased lipid accumulation occurs due to reduced mitochondrial utilization by β-oxidation of FA due to the extensive mitochondrial damage observed in our study. This is further supported by our lipidomic results, whereby accumulated acyl-CoA not utilized by mitochondria may provide a substrate for the enzymatic synthesis of new lipid molecules (e.g., diacylglycerols, triacylglycerols, and others) that we observe. Prolonged exposure to ISO increased intracellular DAG, lysophosphatidylcholine (LPC 18:0), triacylglycerols (TG), and phosphatidylethanolamines (PE), whereas intracellular phosphatidylcholine (PC) showed a mixed effect. Importantly, analysis of the differentially affected lipid classes provides insight into the possible functional consequences of their accumulation and their role in the pathogenesis of TTS. Our results show increased TG accumulation, which was previously unknown in a TTS model. There was also a study where serum TG levels were increased in TTS patients (48). This accumulation is likely due to mitochondrial dysfunction, which has already been observed in mouse preadipocytes (35) as well as in the present study. In several heart failure models, it has been observed that increased TG levels lead to impaired cardiomyocyte contractility (49, 50). Thus, it is very likely that the increased amount of TG observed in our TTS model contributes to the development of left ventricular hypokinesis in TTS patients. Many other lipotoxic molecules may control the pathogenesis of TTS observed in our study. The lipidomic spectral analysis confirmed increased levels of the signaling lipid molecules DAG, LPC, and PE. All of these lipids can lead to the activation of protein kinase C (PKC) (22, 51, 52), which leads to the activation of another kinase, mitogen-activated protein kinase (MAPK) (21), that regulates the cell nucleus. Up-regulation of this pathway has been frequently detected in cardiac remodeling (53), apoptosis (33), and hypertrophy (22).

Increased DAG, including increased lipid accumulation, has already been observed in a mouse model of TTS (9). Another study also confirmed increased MAPK activation in a rat model of TTS on the cardiomyocyte cell line H9C2 (34, 35). Thus, it is very likely that the accumulation of DAG, LPC, and PE observed in the present study has a major contribution to both contractile dysfunction and cardiomyocyte apoptosis and to increasing the risk of later development of heart failure. A decrease in lysophosphatidylethanolamines (LPE), a molecule derived from the partial hydrolysis of PE and about which we know little, was also observed. Changes in sphingomyelin (SM) content had a mixed effect. Both increased production and increased degradation of SM caused changes in cellular pathways. SM degradation leads to ceramide production with proven proapoptotic effects (54).

On the other hand, higher levels of SM associated with inflammation have been observed in coronary artery smooth muscle cells (55). Given that both inflammation and apoptosis have been observed in TTS, it can be hypothesized that changes in SM content could play a role in the development of inflammation and apoptosis during TTS (56, 57). Similarly, phosphatidylinositol, a lipid that can be phosphorylated and is involved in PKC and MAPK activation (58), has a mixed effect. Although conclusive data demonstrating a causal link between elevated toxic lipids and the pathogenesis of TTS are not yet available, our observations provide for the first time a comprehensive view of the induction of lipotoxicity due to mitochondrial damage induced by increased catecholamine stimulation.

Although *in vitro* experiments in HL-1 cells allow for direct investigation of isoprenaline-induced effects on cardiomyocyte metabolic functions without interference from other neuronal and/or endocrine factors present in *in vivo* experiments (whole organism), extrapolation to human pathophysiology needs to be done with care.

### Limitations

First, the isoprenaline concentration employed in cellular TTS models (9, 45), as well as in the present study, are significantly higher than the concentrations of catecholamines observed in the plasma of TTS patients (approximately 2–5.5 nM for epinephrine) (3, 4). However, local concentrations of catecholamines released from cardiac sympathetic efferent neurons are likely to be higher than plasma concentrations. The measured noradrenaline concentration in the aortic root and coronary sinus reached up to 34 nM (three times normal noradrenaline levels) (59). In addition, *in vitro* cultures and media may reduce adrenergic signaling (decreased adrenergic receptors, decreased intracellular signaling, and absence of other endocrine factors with permissive effects). Second, the study of FA oxidation utilized etomoxir as a CPT-1 inhibitor; however, short-chain fatty acids do not require this transport mechanism, even though the intracellular quantity of short-chain FFAs is limited (60). Third, since isoprenaline exposure has been previously shown to reduce cell proliferation and induce apoptosis (61, 62), relevant data were normalized to cell count, total protein, or amount of mRNA. Fourth, HL-1 cells (derived from atrial cardiomyocytes) were used in this study, which contrasts with the primary ventricular location of dysfunction in TTS. However, it should be noted that alternative options, e.g., using neonatal cardiomyocytes, also have considerable limitations since TTS predominantly develops in adults (63). Furthermore, the metabolism of neonatal cardiomyocytes is more glycolytic than the metabolism of adult cardiomyocytes (64), and previous studies also showed that HL-1 cardiomyocytes are phenotypically more stable with better contractility than neonatal rat cardiomyocytes (65–67).

To enhance fatty acid utilization, HL-1 cells were starved for 2 h in a low glucose medium. Although the HL-1-cell line represents a well-established model characterized by morphological, biochemical, and electrophysiological characteristics of differentiated cardiomyocytes (33), it is necessary to note associated limitations.

## Conclusion

Our study showed that prolonged but not acute isoprenaline exposure increased the total lipid content and profoundly modified the intracellular lipid profile with a reduction in cardioprotective molecules and an increase in lipotoxic molecules. In parallel, isoprenaline administration rapidly reduced ATP production (within 70 min) with a subsequent reduction in β-oxidation after prolonged exposure. The results of this study provide possible molecular explanations for the pathogenesis of TTS based on increased mitochondrial dysfunction (presumably due to ROS production), i.e., reduced β-oxidation → accumulation of lipotoxic molecules → impaired contractility/apoptosis/remodeling. Furthermore, the provided hypothesis can also help explain progression to heart failure as a long-term consequence of TTS since accumulated lipids might exert prolonged effects on cellular signaling *via* the MAPK and PKC pathways. Clearly, further research in the area is warranted, particularly with a focus on the role of cardiomyocyte lipolysis, fatty acid uptake, and the effects of individual lipotoxic molecules on cardiomyocyte function.

## Data availability statement

The original contributions presented in this study are included in the article/Supplementary material, further inquiries can be directed to the corresponding author.

## Author contributions

JP and PT conceived the project, designed the experiments, and reviewed and edited this manuscript. IF supervised all experiments and wrote the manuscript. IF, MT, LV, MH, ME, and SM performed experiments and data analysis. OF performed data analyses. KB, VK, and JH performed the lipidomic analysis. IF, JP, and PT acquired funding for the project. All authors have read and agreed to the published version of the manuscript.
