# Supplementary material for: Isoprenaline modified the lipidomic profile and reduced β-oxidation in HL-1 cardiomyocytes: In vitro model of takotsubo syndrome
Source: Front Cardiovasc Med. 2022 Aug 22;9:917989. doi: 10.3389/fcvm.2022.917989 (PMC9441769; doi:10.3389/fcvm.2022.917989)
Supplement: Supplementary file 1 [file Data_Sheet_1.docx]

Supplementary Material

# Supplementary Methods

## Details of lipidomic analysis

Lipids were extracted in mixture of methyl tert-butyl ether and methanol (10:3). Subsequently, deionized water was added, samples were centrifuged (14.000 rpm, 10 min) and evaporated to dryness. Samples were then reconstituted in mixture of isopropanol, methanol and water (65:30:5). For the lipidomic analysis, U-HPLC (Infinity 1290, Agilent, USA) coupled to a high-resolution mass spectrometer with a hyphenated quadrupole time-of-flight mass analyzer (6560 Ion Mobility Q-TOF LC/MS; Agilent, USA) with the Agilent Jet Stream electrospray source were employed. An Acquity BEH C18 (1.7 μm, 2.1 mm x 150 mm (Waters, USA)) was used for chromatographic separation. The chromatographic system used with ESI+ detection was: (A) - 10 mM ammonium formate and 0.1% formic acid in acetonitrile: water (60:40, v/v); (B) – 10 mM ammonium formate and 0.1% formic acid in 2-propanol:acetonitrile (90:10, v/v). For chromatographic separation of plasma detected in ESI- mode, following mobile phases were used: (A) - 10 mM ammonium acetate and 0.1% acetic acid in acetonitrile: water (60:40); (B) - 10 mM ammonium acetate and 0.1% acetic acid in 2-propanol: acetonitrile (90:10, v/v). The flow rate was constant at 0.300 ml/min. The column temperature was maintained at 60 °C, the injection volume was increased to 1 μl in ESI+ mode and 5 μl in ESI- mode. The autosampler was kept at 10 °C. A QC sample was run every 10 samples for system stability assessment. The samples were measured in MS mode, the fragmentation experiments were run on the QC sample at the end of the batch at 10, 20 and 40 Ev. The Agilent 6560 Ion Mobility mass analyzer was operated at following conditions at both ionization modes: Gas temperature 350 °C, Drying Gas 12 l/min, Nebulizer pressure 40 psig, Sheath gas flow 11 l/min, Nozzle voltage 250 V, fragmentor voltage 380 V, octopole radiofrequency voltage 750 V. The capillary voltage was 3000 V in ESI+, 4000 V in ESI-. For all samples, the data were acquired over the m/z range of 100 – 1700 at the rate of 1 spectrum/s. The m/z range was autocorrected on reference masses 121.0509 and 922.0098 for positive mode and 119.0363 and 980.0164 in negative mode. To obtain the fragmentation spectra of lipids, the QC sample was run several times in auto MS/MS mode with collision energies 10, 20 and 40 eV, with acquisition rate of 5 spectra/s for full spectra and 7 spectra/s for fragmentation spectra. Top 5 ions were chosen for fragmentation with dynamic exclusion for 0.1 min after fragmentation event.

# Supplementary Figures and Tables

## Supplementary Figures


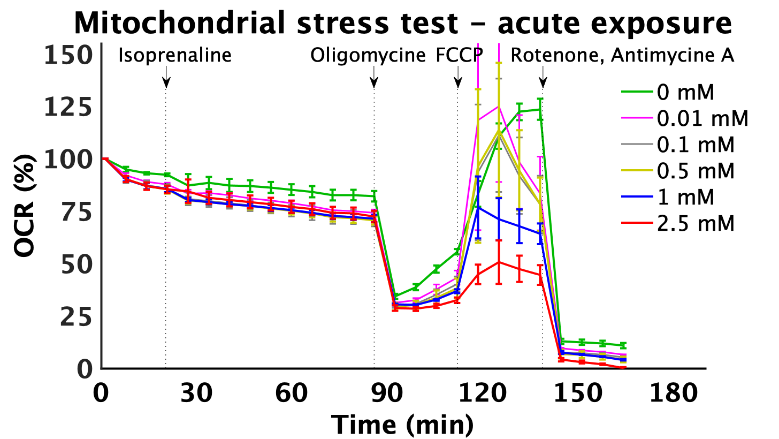

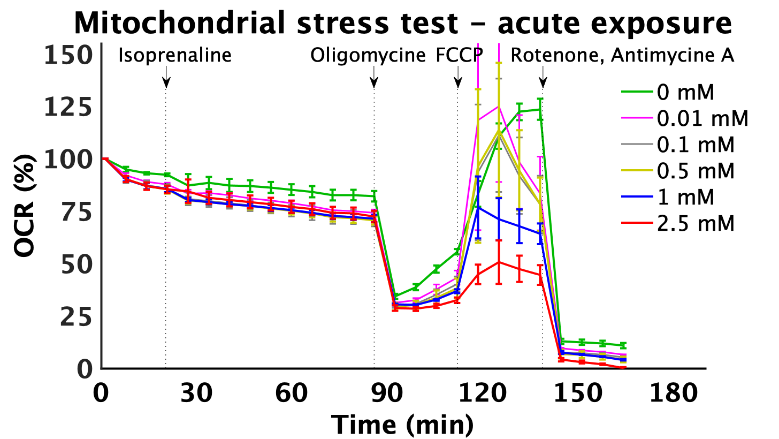


**Supplementary** **Figure 1:** The real-time oxygen consumption rate of HL-1 cardiomyocyte expose to ISO (acute exposure and 24 hours exposure) during mitochondrial stress test.


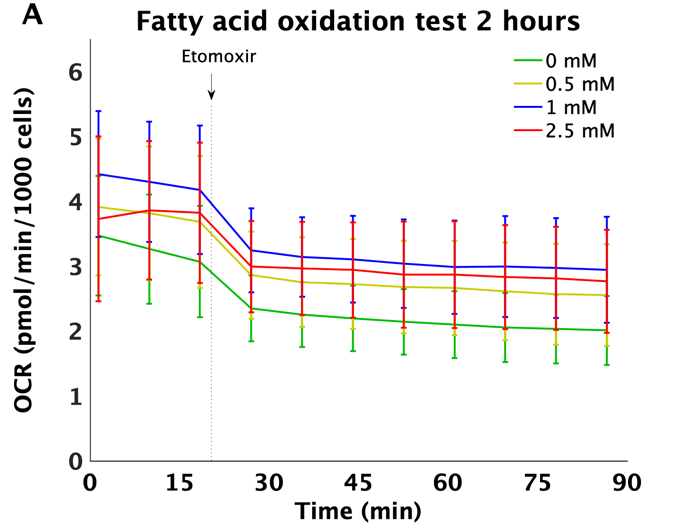

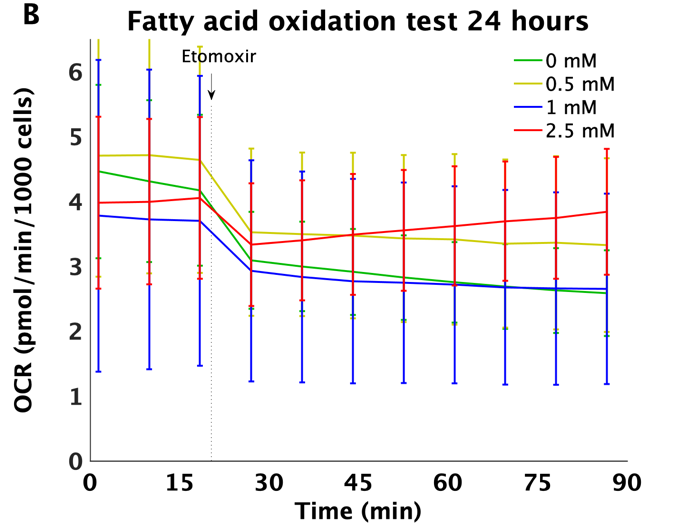


**Supplementary** **Figure 2:** (A) The real-time oxygen consumption rate of HL-1 cardiomyocyte of fatty acid oxidation after 2 hours exposure to ISO. (B) The real-time oxygen consumption rate of HL-1 cardiomyocyte of fatty acid oxidation after 24 hours exposure to ISO.

**
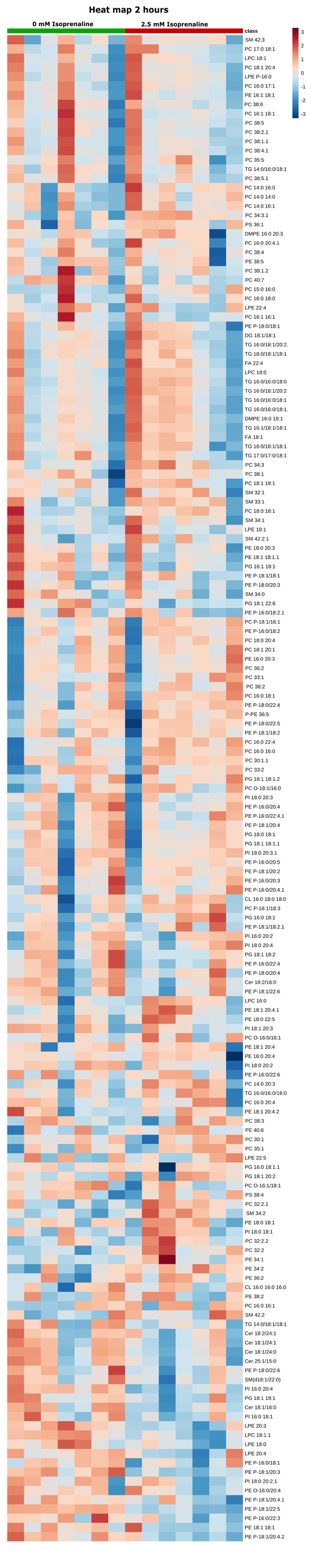

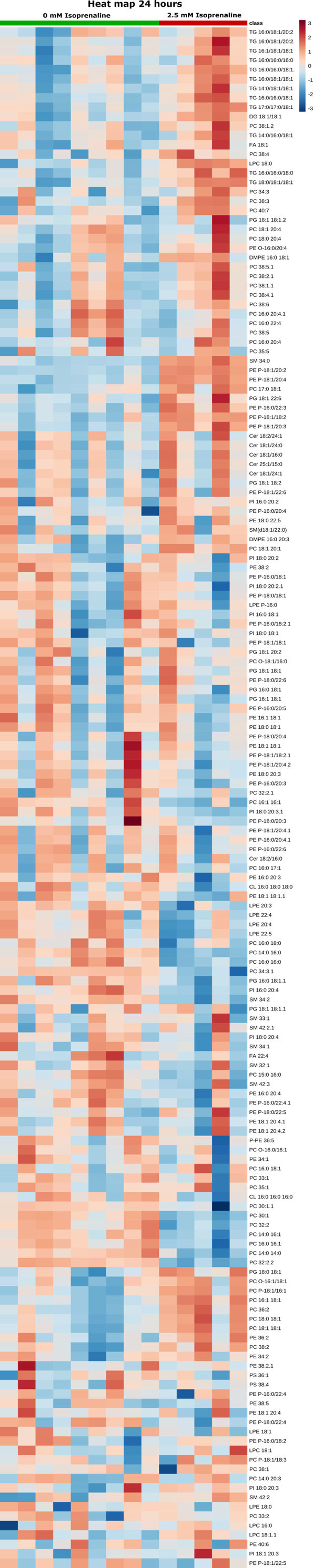
**

**
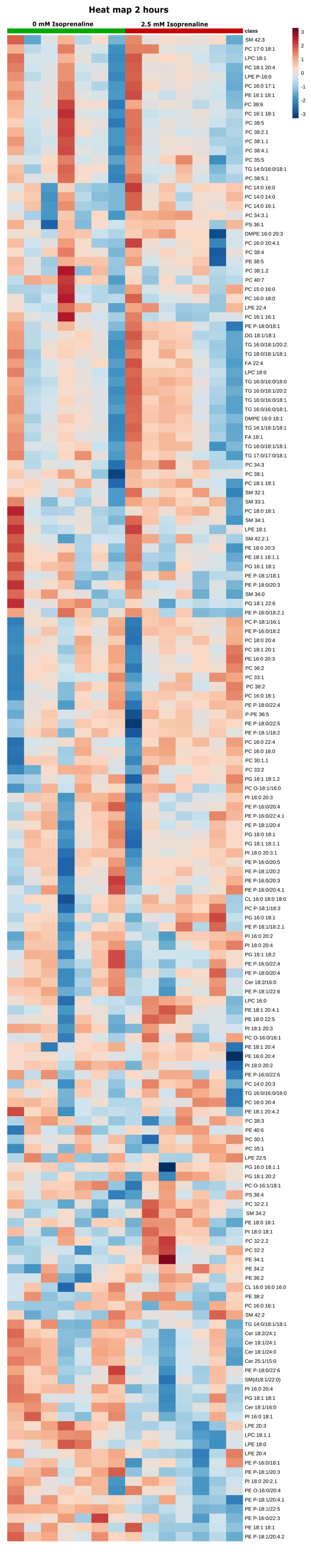

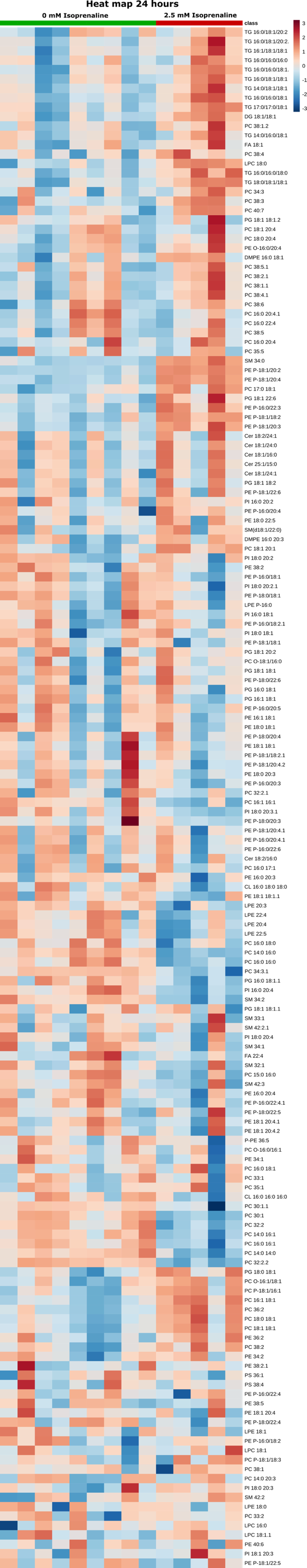
**

**Supplementary** **Figure 3:** (A) Heatmap of all detected lipid molecules in the HL-1 cardiomyocytes expose to 2.5 mM of isoprenaline for 2 hours. (B) Heatmap of all detected lipid molecules in the HL-1 cardiomyocytes expose to 2.5 mM of isoprenaline for 24 hours.

## Supplementary Tables

**Supplementary Table 1:** Lipidomic changes after 2 hours of ISO exposure of all lipids

| **Category** | **Subclass** | **Common name** | **Fold change** | **FDR p value** |
| --- | --- | --- | --- | --- |
| Fatty acyls | Fatty acids | FA 18:1 | 0.95 | 0.99 |
|  |  | FA 22:4 | 1.25 | 0.99 |
|  |  |  |  |  |
| Glycerolipids | Diacylglycerol | DG 18:1/18:1 | 1.14 | 0.99 |
|  |  |  |  |  |
|  | Triacylglycerols | TG 14:0/16:0/18:1 | 1.13 | 0.89 |
|  |  | TG 14:0/18:1/18:1 | 1.09 | 0.99 |
|  |  | TG 16:0/16:0/16:0 | 0.98 | 0.99 |
|  |  | TG 16:0/16:0/18:0 | 0.88 | 0.99 |
|  |  | TG 16:0/16:0/18:1 | 1.44 | 0.96 |
|  |  | TG 16:0/18:1/18:1 | 0.89 | 0.99 |
|  |  | TG 16:0/18:1/18:1 | 0.96 | 0.99 |
|  |  | TG 16:0/18:1/20:2 | 0.95 | 0.99 |
|  |  | TG 16:0/18:1/20:2 | 1.03 | 0.99 |
|  |  | TG 16:1/18:1/18:1 | 1.1 | 0.99 |
|  |  | TG 17:0/17:0/18:1 | 1.11 | 0.99 |
|  |  | TG 18:0/18:1/18:1 | 1.05 | 0.99 |
|  |  |  |  |  |
| Glycerophospholipids | Cardiolipins | CL 16:0/16:0/16:0/18:1 | 0.94 | 0.99 |
|  |  | CL 16:0/18:0/18:0/18:1 | 1.09 | 0.88 |
|  |  |  |  |  |
|  | Dimyristoylglycerophosphoethanolamines | DMPE 16:0/18:1 | 1.13 | 0.99 |
|  |  | DMPE 16:0/20:3 | 0.52 | 0.92 |
|  |  |  |  |  |
|  | Lysophosphatidylcholine | **LPC 16:0** | **1.32** | **0.04** |
|  |  | LPC 18:0 | 1.07 | 0.44 |
|  |  | LPC 18:1 | 1.04 | 0.99 |
|  |  | **LPC 18:1** | **1.54** | **0.04** |
|  |  |  |  |  |
|  | Lysophosphatidylethanolamines | LPE 18:0 | 0 | 0.45 |
|  |  | LPE 18:1 | 0.94 | 0.99 |
|  |  | LPE 20:3 | 0.77 | 0.99 |
|  |  | LPE 20:4 | 0.97 | 0.62 |
|  |  | LPE 22:4 | 0.83 | 0.41 |
|  |  | LPE 22:5 | 0.97 | 0.61 |
|  |  | LPE (P-16:0) | 0.82 | 0.61 |
|  |  |  |  |  |
|  | Phosphatydilcholins | PC 14:0/14:0 | 0.96 | 0.62 |
|  |  | PC 14:0/16:0 | 1.89 | 0.82 |
|  |  | PC 14:0/16:1 | 0.99 | 0.88 |
|  |  | PC 14:0/20:3 | 1.18 | 0.89 |
|  |  | PC 15:0/16:0 | 0.77 | 0.99 |
|  |  | PC 16:0/16:0 | 0.98 | 0.99 |
|  |  | PC 16:0/16:1 | 0.93 | 0.86 |
|  |  | PC 16:0/17:1 | 0.84 | 0.99 |
|  |  | PC 16:0/18:0 | 1.02 | 0.99 |
|  |  | PC 16:0/18:1 | 1.08 | 0.86 |
|  |  | PC 16:0/20:4 | 1.09 | 0.92 |
|  |  | PC 16:0/20:4 | 0.98 | 0.99 |
|  |  | PC 16:0/22:4 | 1.18 | 0.99 |
|  |  | PC 16:1/16:1 | 0.87 | 0.99 |
|  |  | PC 16:1/18:1 | 0.94 | 0.99 |
|  |  | PC 17:0/18:1 | 0.88 | 0.99 |
|  |  | PC 18:0/18:1 | 0.82 | 0.99 |
|  |  | PC 18:0/20:4 | 1.05 | 0.99 |
|  |  | PC 18:1/18:1 | 0.9 | 0.99 |
|  |  | PC 18:1/20:1 | 0.96 | 0.99 |
|  |  | PC 18:1/20:4 | 0.88 | 0.99 |
|  |  |  |  |  |
|  | Phosphatidylethanolamines | PE 16:0/20:3 | 1.01 | 0.99 |
|  |  | PE 16:0/20:4 | 1.29 | 0.99 |
|  |  | PE 16:1/18:1 | 1.03 | 0.99 |
|  |  | PE 18:0/18:1 | 1.02 | 0.61 |
|  |  | PE 18:0/20:3 | 1.03 | 0.72 |
|  |  | PE 18:0/20:4 | 1.72 | 0.96 |
|  |  | PE 18:0/20:4 | 0.95 | 0.53 |
|  |  | PE 18:0/22:5 | 0 | 0.99 |
|  |  | PE 18:1/18:1 | 0.98 | 0.99 |
|  |  | PE 18:1/18:1 | 0.89 | 0.89 |
|  |  | PE 18:1/20:4 | 0.97 | 0.81 |
|  |  | PE (P-16:0/18:1) | 0.81 | 0.99 |
|  |  | PE (P-16:0/18:2) | 0.97 | 0.99 |
|  |  | PE (P-16:0/18:2) | 0.98 | 0.99 |
|  |  | PE (P-16:0/20:3) | 0.92 | 0.99 |
|  |  | PE (P-16:0/20:4) | 1.28 | 0.44 |
|  |  | PE (P-16:0/20:4) | 0.99 | 0.8 |
|  |  | PE (P-16:0/20:5) | 1.07 | 0.99 |
|  |  | PE (P-16:0/22:3) | 0.96 | 0.99 |
|  |  | PE (P-16:0/22:4) | 1.03 | 0.88 |
|  |  | PE (P-16:0/22:4) | 1.08 | 0.99 |
|  |  | PE (P-16:0/22:6) | 0.94 | 0.99 |
|  |  | PE (P-18:0/18:1) | 0.66 | 0.99 |
|  |  | PE (P-18:0/20:3) | 0.77 | 0.99 |
|  |  | PE (P-18:0/20:4) | 0.95 | 0.59 |
|  |  | PE (P-18:0/22:4) | 0.81 | 0.99 |
|  |  | PE (P-18:0/22:5) | 0.65 | 0.99 |
|  |  | PE (P-18:0/22:6) | 1.04 | 0.99 |
|  |  | PE (P-18:1/18:1) | 1.08 | 0.99 |
|  |  | **PE (P-18:1/18:2)** | **1.6** | **0.04** |
|  |  | PE (P-18:1/18:2) | 1.1 | 0.99 |
|  |  | PE (P-18:1/20:2) | 0.98 | 0.3 |
|  |  | PE (P-18:1/20:3) | 1.51 | 0.06 |
|  |  | **PE (P-18:1/20:4)** | **1.86** | **1.2 x 10^-4^** |
|  |  | PE (P-18:1/20:4) | 0.77 | 0.96 |
|  |  | PE (P-18:1/20:4) | 1.05 | 0.67 |
|  |  | PE (P-18:1/22:5) | 0.92 | 0.99 |
|  |  | PE (P-18:1/22:6) | 0.86 | 0.67 |
|  |  | PE 34:1 | 0.61 | 0.53 |
|  |  | PE 34:2 | 0.68 | 0.32 |
|  |  | PE 36:2 | 0.92 | 0.77 |
|  |  | PE 38:2 | 0.64 | 0.99 |
|  |  | PE 38:5 | 1.06 | 0.99 |
|  |  | PE 40:6 | 1 | 0.67 |
|  |  | PE 36:5 | 0.91 | 0.99 |
|  |  |  |  |  |
|  | Phosphatidylglycerols | PG 16:0/18:1 | 1.17 | 0.99 |
|  |  | PG 16:0/18:1 | 1.11 | 0.45 |
|  |  | PG 16:1/18:1 | 0.96 | 0.99 |
|  |  | PG 18:0/18:1 | 0 | 0.99 |
|  |  | PG 18:1/18:1 | 1.41 | 0.91 |
|  |  | PG 18:1/18:1 | 1.02 | 0.99 |
|  |  | PG 18:1/18:1 | 0.91 | 0.53 |
|  |  | PG 18:1/18:2 | 0.35 | 0.53 |
|  |  | PG 18:1/20:2 | 0.97 | 0.99 |
|  |  | **PG 18:1/22:6** | **1.59** | **0.04** |
|  |  |  |  |  |
|  | Phosphatidylinositols | PI 16:0/18:1 | 0.88 | 0.99 |
|  |  | PI 16:0/20:2 | 1.27 | 0.44 |
|  |  | PI 16:0/20:4 | 1.18 | 0.89 |
|  |  | PI 18:0/18:1 | 1 | 0.82 |
|  |  | PI 18:0/20:2 | 1.1 | 0.99 |
|  |  | PI 18:0/20:2 | 1.04 | 0.99 |
|  |  | PI 18:0/20:3 | 1 | 0.67 |
|  |  | PI 18:0/20:3 | 0.98 | 0.99 |
|  |  | PI 18:0/20:4 | 0.91 | 0.99 |
|  |  | PI 18:1/20:3 | 1.04 | 0.72 |
|  |  |  |  |  |
|  | Phosphatydilcholins | PC (P-18:1/16:1) | 1.03 | 0.99 |
|  |  | PC (P-18:1/18:3) | 1.06 | 0.92 |
|  |  | PC (O-16:0/16:1) | 0.89 | 0.99 |
|  |  | PC (O-16:0/20:4) | 0.8 | 0.99 |
|  |  | PC (O-16:1/18:1) | 2.54 | 0.99 |
|  |  | PC (O-18:1/16:0) | 0.99 | 0.68 |
|  |  | PC 30:1 | 0.86 | 0.99 |
|  |  | PC 30:1 | 0.93 | 0.99 |
|  |  | PC 32:2 | 0.8 | 0.24 |
|  |  | PC 32:2 | 0.72 | 0.53 |
|  |  | PC 32:2 | 0.94 | 0.68 |
|  |  | PC 33:1 | 1.14 | 0.99 |
|  |  | PC 33:2 | 0.94 | 0.99 |
|  |  | PC 34:3 | 0.55 | 0.53 |
|  |  | PC 34:3 | 1.18 | 0.22 |
|  |  | PC 35:1 | 0.93 | 0.99 |
|  |  | PC 35:5 | 1.1 | 0.99 |
|  |  | PC 36:2 | 1.06 | 0.99 |
|  |  | PC 38:1 | 1.25 | 0.92 |
|  |  | PC 38:1 | 0.9 | 0.99 |
|  |  | PC 38:1 | 0.95 | 0.99 |
|  |  | PC 38:2 | 1.06 | 0.99 |
|  |  | PC 38:2 | 0.68 | 0.99 |
|  |  | PC 38:3 | 0.97 | 0.99 |
|  |  | PC 38:4 | 1.29 | 0.99 |
|  |  | PC 38:4 | 0.97 | 0.99 |
|  |  | PC 38:5 | 0.9 | 0.99 |
|  |  | PC 38:5 | 0.94 | 0.99 |
|  |  | PC 38:6 | 1.09 | 0.99 |
|  |  | PC 40:7 | 0.99 | 0.67 |
|  |  |  |  |  |
|  | Phosphatidylserines | PS 36:1 | 1.04 | 0.79 |
|  |  | PS 38:4 | 0.99 | 0.99 |
|  |  |  |  |  |
| Sphingolipids | Ceramides | Cer 18:1/16:0 | 0.95 | 0.24 |
|  |  | Cer 18:1/24:0 | 1.35 | 0.44 |
|  |  | Cer 18:1/24:1 | 0.93 | 0.53 |
|  |  | Cer 18:2/16:0 | 1.28 | 0.96 |
|  |  | Cer 18:2/24:1 | 1.67 | 0.93 |
|  |  | Cer 25:1/15:0 | 0.84 | 0.44 |
|  |  |  |  |  |
|  | Sphingomyelins | SM 32:1 | 1.35 | 0.99 |
|  |  | SM 33:1 | 0.93 | 0.67 |
|  |  | SM 34:0 | 2.73 | 0.99 |
|  |  | SM 34:1 | 1.08 | 0.99 |
|  |  | SM 34:2 | 0.98 | 0.24 |
|  |  | SM 42:2 | 0.96 | 0.72 |
|  |  | SM 42:2 | 1.15 | 0.96 |
|  |  | SM 42:3 | 1.16 | 0.99 |
|  |  | SM d18:1/22:0 | 1.07 | 0.99 |

**Supplementary Table 2:** Lipidomic changes after 24 hours of ISO exposure of all lipids

| **Category** | **Subclass** | **Common name** | **Fold change** | **FDR p value** |
| --- | --- | --- | --- | --- |
| Fatty acyls | Fatty acids | FA 18:1 | 1.09 | 0.23 |
|  |  | FA 22:4 | 0.98 | 0.51 |
|  |  |  |  |  |
| Glycerolipids | Diacylglycerol | **DG 18:1/18:1** | **1.34** | **0.01** |
|  |  |  |  |  |
|  | Triacylglycerols | TG 14:0/16:0/18:1 | 1.12 | 0.24 |
|  |  | **TG 14:0/18:1/18:1** | **1.15** | **0.05** |
|  |  | TG 16:0/16:0/16:0 | 1.09 | 0.36 |
|  |  | **TG 16:0/16:0/18:0** | **1.35** | **0.01** |
|  |  | **TG 16:0/16:0/18:1** | **1.26** | **0.01** |
|  |  | **TG 16:0/18:1/18:1** | **1.44** | **0.04** |
|  |  | TG 16:0/18:1/18:1 | 1.21 | 0.1 |
|  |  | TG 16:0/18:1/20:2 | 1.5 | 0.45 |
|  |  | TG 16:0/18:1/20:2 | 1.19 | 0.07 |
|  |  | TG 16:1/18:1/18:1 | 1.26 | 0.08 |
|  |  | **TG 17:0/17:0/18:1** | **1.59** | **0.02** |
|  |  | **TG 18:0/18:1/18:1** | **1.78** | **0.01** |
|  |  |  |  |  |
| Glycerophospholipids | Cardiolipins | CL 16:0/16:0/16:0/18:1 | 0.67 | 0.11 |
|  |  | CL 16:0/18:0/18:0/18:1 | 0.83 | 0.11 |
|  |  |  |  |  |
|  | Dimyristoylglycerophosphoethanolamines | DMPE 16:0/18:1 | 1.22 | 0.1 |
|  |  | DMPE 16:0/20:3 | 1.24 | 0.28 |
|  |  |  |  |  |
|  | Lysophosphatidylcholine | LPC 16:0 | 1.03 | 0.81 |
|  |  | **LPC 18:0** | **1.48** | **2.49 x10^-3^** |
|  |  | LPC 18:1 | 1.03 | 0.51 |
|  |  | LPC 18:1 | 1.11 | 0.34 |
|  |  |  |  |  |
|  | Lysophosphatidylethanolamines | LPE 18:0 | 0.99 | 0.83 |
|  |  | LPE 18:1 | 0.87 | 0.46 |
|  |  | **LPE 20:3** | **0.13** | **0.01** |
|  |  | LPE 20:4 | 0.64 | 0.11 |
|  |  | LPE 22:4 | 0.4 | 0.13 |
|  |  | **LPE 22:5** | **0.48** | **0.04** |
|  |  | LPE (P-16:0) | 1.06 | 0.98 |
|  |  |  |  |  |
|  | Phosphatydilcholins | **PC 14:0/14:0** | **0.77** | **1.9 x10^-3^** |
|  |  | **PC 14:0/16:0** | **0.54** | **3.3 x10^-5^** |
|  |  | **PC 14:0/16:1** | **0.74** | **2.84 x10^-3^** |
|  |  | PC 14:0/20:3 | 0.67 | 0.97 |
|  |  | **PC 15:0/16:0** | **0.67** | **0.06** |
|  |  | **PC 16:0/16:0** | **0.57** | **2.53 x10^-3^** |
|  |  | **PC 16:0/16:1** | **0.63** | **4.41 x10^-3^** |
|  |  | PC 16:0/17:1 | 0.82 | 0.78 |
|  |  | PC 16:0/18:0 | 0.83 | 0.12 |
|  |  | PC 16:0/18:1 | 1.07 | 0.85 |
|  |  | PC 16:0/20:4 | 1.03 | 0.58 |
|  |  | PC 16:0/20:4 | 0.97 | 0.72 |
|  |  | PC 16:0/22:4 | 1.02 | 0.97 |
|  |  | PC 16:1/16:1 | 0.66 | 0.03 |
|  |  | **PC 16:1/18:1** | **1.59** | **2.1 x 10^-3^** |
|  |  | **PC 17:0/18:1** | **1.23** | **0.01** |
|  |  | **PC 18:0/18:1** | **1.31** | **0.02** |
|  |  | PC 18:0/20:4 | 1.06 | 0.45 |
|  |  | **PC 18:1/18:1** | **1.39** | **0.02** |
|  |  | **PC 18:1/20:1** | **1.27** | **0.01** |
|  |  | PC 18:1/20:4 | 0.96 | 0.85 |
|  |  | PC (O-16:0/16:1) | 0.93 | 0.28 |
|  |  | PC (O-16:0/20:4) | 1.24 | 0.15 |
|  |  | PC (O-16:1/18:1) | 1.34 | 0.14 |
|  |  | PC (O-18:1/16:0) | 1.05 | 0.72 |
|  |  | **PC (P-18:1/16:1)** | **1.69** | **0.04** |
|  |  | PC (P-18:1/18:3) | 1.44 | 0.52 |
|  |  | **PC 30:1** | **0.6** | **3.65 x10^-4^** |
|  |  | **PC 30:1** | **0.6** | **0.05** |
|  |  | **PC 32:2 1** | **0.73** | **2.1 x10^-3^** |
|  |  | PC 32:2 | 0.8 | 0.51 |
|  |  | **PC 32:2 2** | **0.71** | **2.1 x10^-3^** |
|  |  | PC 33:1 | 0.96 | 0.75 |
|  |  | PC 33:2 | 0.91 | 0.89 |
|  |  | PC 34:3 | 1.1 | 0.21 |
|  |  | **PC 34:3** | **0.6** | **1.69 x10^-3^** |
|  |  | PC 35:1 | 1.05 | 0.97 |
|  |  | PC 35:5 | 1 | 0.94 |
|  |  | **PC 36:2** | **1.53** | **0.01** |
|  |  | PC 38:1 | 1.17 | 0.97 |
|  |  | PC 38:1 | 1.18 | 0.25 |
|  |  | PC 38:1 | 1.21 | 0.07 |
|  |  | PC 38:2 | 1.21 | 0.11 |
|  |  | PC 38:2 | 1.23 | 0.24 |
|  |  | **PC 38:3** | **1.29** | **0.04** |
|  |  | PC 38:4 | 1.17 | 0.1 |
|  |  | PC 38:4 | 1.26 | 0.24 |
|  |  | PC 38:5 | 1.02 | 0.83 |
|  |  | PC 38:5 | 1.19 | 0.31 |
|  |  | PC 38:6 | 1.08 | 0.63 |
|  |  | PC 40:7 | 1.12 | 0.11 |
|  |  |  |  |  |
|  | Phosphatidylethanolamines | PE 16:0/20:3 | 0.92 | 0.3 |
|  |  | PE 16:0/20:4 | 0.78 | 0.21 |
|  |  | PE 16:1/18:1 | 0.96 | 0.65 |
|  |  | PE 18:0/18:1 | 0.97 | 0.85 |
|  |  | PE 18:0/20:3 | 0.85 | 0.44 |
|  |  | PE 18:0/20:4 | 0.72 | 0.83 |
|  |  | PE 18:0/20:4 | 0.86 | 0.73 |
|  |  | PE 18:0/22:5 | 1.09 | 0.51 |
|  |  | PE 18:1/18:1 | 1.04 | 0.18 |
|  |  | PE 18:1/18:1 | 0.58 | 0.94 |
|  |  | PE 18:1/20:4 | 0.88 | 0.76 |
|  |  | PE (P-16:0/18:1) | 0.79 | 0.35 |
|  |  | PE (P-16:0/18:2) | 1.09 | 0.83 |
|  |  | PE (P-16:0/18:2) | 1.14 | 0.89 |
|  |  | PE (P-16:0/20:3) | 0.76 | 0.21 |
|  |  | PE (P-16:0/20:4) | 1.18 | 0.24 |
|  |  | PE (P-16:0/20:4) | 0.93 | 0.89 |
|  |  | PE (P-16:0/20:5) | 1.1 | 0.97 |
|  |  | **PE (P-16:0/22:3)** | **1.27** | **2.52 x10^-3^** |
|  |  | PE (P-16:0/22:4) | 0.94 | 0.18 |
|  |  | PE (P-16:0/22:4) | 0.8 | 0.61 |
|  |  | PE (P-16:0/22:6) | 0.85 | 0.81 |
|  |  | PE (P-18:0/18:1) | 0.71 | 0.45 |
|  |  | PE (P-18:0/20:3) | 0.81 | 0.44 |
|  |  | PE (P-18:0/20:4) | 0.91 | 0.97 |
|  |  | PE (P-18:0/22:4) | 0.81 | 0.21 |
|  |  | PE (P-18:0/22:5) | 1 | 0.77 |
|  |  | PE (P-18:0/22:6) | 1 | 0.45 |
|  |  | PE (P-18:1/18:1) | 0.74 | 0.25 |
|  |  | **PE (P-18:1/18:2)** | **2.16** | **1.69 x10^-3^** |
|  |  | PE (P-18:1/18:2) | 0.91 | 0.76 |
|  |  | **PE (P-18:1/20:2)** | **2.74** | **3.04 x10^-7^** |
|  |  | **PE (P-18:1/20:3)** | **1.65** | **5.56 x10^-4^** |
|  |  | **PE (P-18:1/20:4)** | **3.88** | **1.75 x10^-5^** |
|  |  | PE (P-18:1/20:4) | 0.87 | 0.6 |
|  |  | PE (P-18:1/20:4) | 0.81 | 0.34 |
|  |  | PE (P-18:1/22:5) | 0.63 | 0.81 |
|  |  | PE (P-18:1/22:6) | 1 | 0.46 |
|  |  | PE 34:1 | 0.9 | 0.17 |
|  |  | PE 34:2 | 1.07 | 0.59 |
|  |  | PE 36:2 | 1.14 | 0.11 |
|  |  | PE 38:2 | 0.46 | 0.54 |
|  |  | PE 38:5 | 0.82 | 0.85 |
|  |  | PE 40:6 | 0.97 | 0.89 |
|  |  | PE 36:5 | 0.98 | 0.36 |
|  |  |  |  |  |
|  | Phosphatidylglycerols | PG 16:0/18:1 | 0.92 | 0.88 |
|  |  | **PG 16:0/18:1** | **0.67** | **0.01** |
|  |  | PG 16:1/18:1 | 0.74 | 0.46 |
|  |  | PG 18:0/18:1 | 1.53 | 0.06 |
|  |  | PG 18:1/18:1 | 0.94 | 0.81 |
|  |  | PG 18:1/18:1 | 1.19 | 0.89 |
|  |  | PG 18:1/18:1 | 0.92 | 0.7 |
|  |  | PG 18:1/18:2 | 0.85 | 0.51 |
|  |  | PG 18:1/20:2 | 1.1 | 0.51 |
|  |  | **PG 18:1/22:6** | **1.38** | **0.02** |
|  |  |  |  |  |
| Glycerophospholipids | Phosphatidylinositols | PI 16:0/18:1 | 0.88 | 0.83 |
|  |  | PI 16:0/20:2 | 1.15 | 0.45 |
|  |  | **PI 16:0/20:4** | **0.77** | **0.01** |
|  |  | PI 18:0/18:1 | 0.93 | 0.93 |
|  |  | PI 18:0/20:2 | 1.01 | 0.94 |
|  |  | PI 18:0/20:2 | 0.54 | 0.51 |
|  |  | **PI 18:0/20:3** | **1.32** | **0.04** |
|  |  | PI 18:0/20:3 | 0.59 | 0.45 |
|  |  | PI 18:0/20:4 | 0.91 | 0.45 |
|  |  | PI 18:1/20:3 | 1 | 0.45 |
|  |  |  |  |  |
|  | Phosphatidylserines | PS 36:1 | 1 | 0.58 |
|  |  | PS 38:4 | 1.03 | 0.76 |
|  |  |  |  |  |
| Sphingolipids | Ceramides | Cer 18:1/16:0 | 1 | 0.33 |
|  |  | Cer 18:1/24:0 | 0.99 | 0.32 |
|  |  | Cer 18:1/24:1 | 0.96 | 0.34 |
|  |  | Cer 18:2/16:0 | 0.65 | 0.91 |
|  |  | Cer 18:2/24:1 | 0.95 | 0.51 |
|  |  | Cer 25:1/15:0 | 1.07 | 0.3 |
|  |  |  |  |  |
|  | Sphingomyelins | SM 32:1 | 0.91 | 0.43 |
|  |  | SM 33:1 | 1 | 0.98 |
|  |  | **SM 34:0** | **2.54** | **6.66 x10^-6^** |
|  |  | SM 34:1 | 0.98 | 0.43 |
|  |  | **SM 34:2** | **0.78** | **3.65 x10^-4^** |
|  |  | SM 42:2 | 1.1 | 0.33 |
|  |  | SM 42:2 | 0.97 | 0.78 |
|  |  | SM 42:3 | 0.8 | 0.25 |
|  |  | SM d18:1/22:0 | 1.08 | 0.49 |

**
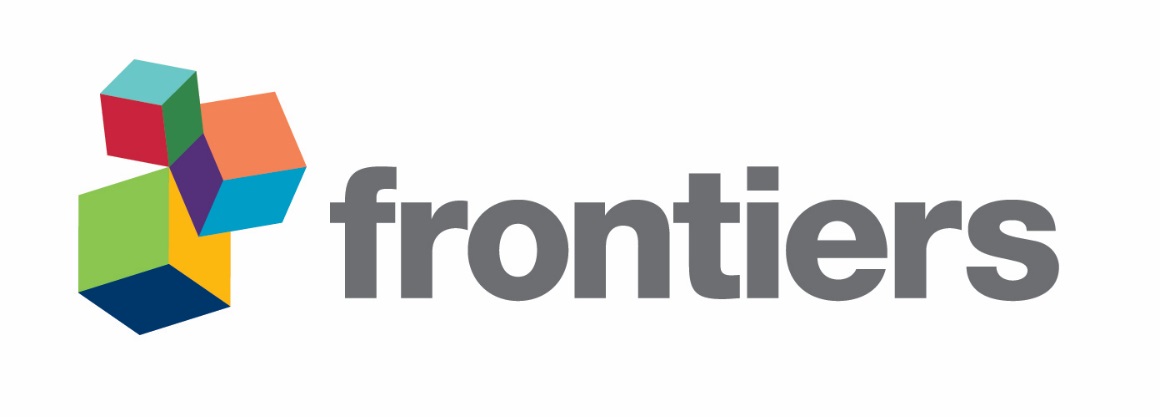
**
